# 553. Nontuberculous Mycobacteria (NTM) Infections in Burn Patients: Risk Factors, Impacts on Recovery, and Outcomes

**DOI:** 10.1093/jbcr/irag033.293

**Published:** 2026-04-06

**Authors:** Janie Faris, Jordan Wartell, Rebecca Coffey, Samuel P Mandell

**Affiliations:** Parkland Health, Grapevine, TX; University of Texas Southwestern Medical Center, Dallas, TX; Parkland Health, Dallas, TX; University of Texas Southwestern / Parkland Health, Dallas, TX

## Abstract

**Introduction:**

Infections in burn patients rise with the TBSA (total body surface area), which also increases morbidity and mortality. Nontuberculous mycobacteria (NTM) are not common (< 1%) in burn patients, with only three cases documented in the literature. Evaluate the rate of NTM infections in burn patients treated at a regional burn center. Secondary outcomes included AFB risk factors, hospital and ICU length, cost, and mortality.

**Methods:**

This is a matched, retrospective cohort study of burn patients admitted from 2015 to 2025. Subjects with a positive culture for NTM were matched to subjects that did not test positive for NTM in a 2:1 manner based on TBSA, age, and sex. Baseline demographics, burn injury data, risk factors for NTM, and microbiological data were collected. Chi Square or Fisher’s Exact was utilized for nominal data and Mann Whitney U for continuous variables. A *p* value of <0.05 was considered significant.

**Results:**

In the study period, 11 subjects with NTM were matched to 20 controls (Table 1). The median time from admission to positive NTM culture was 54 days. There was no difference in time to first excision, but the median time from admission to complete grafting was higher in the NTM group (73 vs 39.5 days). There was no difference between the two groups when comparing the known risk factors for NTM infections. The majority of NTM was *Mycobacterium abscessus* complex (8 subjects) and sites of infection included blood, soft tissue, bone, and lung. Additionally, invasive fungal infections were higher in NTM subjects (9 vs 4 subjects). Hospital costs were significantly higher in the NTM group.

**Conclusions:**

Patients with NTM infections experienced longer, more complicated hospital stays but no difference in mortality. The NTM group did not demonstrate risk factors typically associated with infection, and the late onset of NTM and longer wound closure suggest a nosocomial source.

**Applicability of Research to Practice:**

This infection is rare and can increase morbidity and mortality. With increasing rates of bacterial resistance NTM infections poses a unique risk for burn patients as treatment requires multiple drugs and duration of therapy is up to 6 months of therapy.

**Funding for the study:**

N/A.

